# Effects of Self-Control on Subjective Well-Being: Examining the Moderating Role of Trait and State Motivation

**DOI:** 10.3389/fpsyg.2021.774148

**Published:** 2022-01-10

**Authors:** Guojun Zhao, Fusen Xie, Yuchen Luo, Yixuan Liu, Yuan Chong, Qi Zhang, Wenjie Wang

**Affiliations:** ^1^College of Psychology, Northwest Normal University, Lanzhou, China; ^2^Academy of Plateau Science and Sustainability, Qinghai Normal University, Xining, China; ^3^College of Tourism, Northwest Normal University, Lanzhou, China

**Keywords:** trait self-control, state self-control, subjective well-being, ego depletion, promotion motivation, prevention motivation

## Abstract

It is well documented that self-control has a positive effect on individuals’ subjective well-being. However, little research has focused on the moderators underlying this relationship. The present research used two studies to examine the moderating role of both trait and state motivation on the relationship between self-control and subjective well-being using psychometric and experimental models, respectively. In Study 1, we explored whether trait motivation (including promotion vs. prevention motivation) moderated the relationship between trait self-control and subjective well-being using a psychometric model. In Study 2, we examined the moderating effects of both trait and state motivation on the effect of state self-control (measured via ego depletion) on subjective well-being using an experimental model. Our results indicated that self-control had a positive effect on subjective well-being, with this relationship being primarily moderated by prevention motivation. When state and trait prevention motivations were congruent, self-control had the most obvious impact on subjective well-being. This study suggests that current understandings around the association between self-control and happiness is limited, implying that motivation should be the focus of future research.

## Introduction

Subjective well-being refers to the overall evaluation and feelings of an individual regarding their life (Diener et al., 1985). It is a comprehensive assessment that includes a person’s affective experiences and cognitive evaluation, and usually contains three components: life satisfaction, positive affect, and negative affect (Diener et al., 1985, 2018). With the emergence of positive psychology, subjective well-being has become a representative indicator of an individual’s adaptation to their life. Recently, the association between self-control and subjective well-being has attracted increased attention from researchers (De Ridder et al., 2012; Cheung et al., 2014; Hofmann et al., 2014; Layton and Muraven, 2014; Carter et al., 2015; Grund et al., 2015; Ouyang et al., 2015; Wiese et al., 2018; Fritz and Gallagher, 2019; Nielsen et al., 2019; Joshanloo et al., 2020; Massar et al., 2020; Zeng and Chen, 2020). Self-control is the overriding or inhibiting of automatic, habitual, or innate behaviors, urges, emotions, or desires that would otherwise interfere with one’s goal-directed behaviors (Baumeister et al., 1994; Barkley, 1997). It is regarded as a key variable affecting individuals’ subjective well-being (Hofmann et al., 2014) because it is beneficial in helping people to overcome any experienced interferences, to adhere to their goals, and in motivating themselves to better adapt to life (De Ridder et al., 2012); that is, it helps people to acquire more opportunities to experience happiness. Studies have found that self-control was positively associated with life satisfaction and positive affect, with it being negatively associated with negative affect (Cheung et al., 2014; Grund et al., 2015; Wiese et al., 2018; Nielsen et al., 2019; Massar et al., 2020). However, some researchers have questioned this relationship, arguing that excessive self-control limits an individual’s ability to experience happiness (Zabelina et al., 2007; Layton and Muraven, 2014). They suggest that there may be different types of relationships between self-control and subjective well-being in different circumstances; that is, there are potential moderating factors that may have an effect on this relationship.

The current mainstream view advocates for a better understanding of the nature of self-control from a goal-orientation perspective (Hagger, 2014; Wiese et al., 2018). For example, why do individuals with high self-control report higher subjective well-being? De Ridder and Gillebaart (2017) argued that this could be due to their competence in inhibiting their temptations, with them then being better at initiating goal-directed behaviors. Individuals are more satisfied with their lives and experience more positive affect and less negative affect when they successfully achieve their goals; consequently, an individual’s subjective well-being improves. However, there are different kinds of distractions and temptations involved in the process of pursuing one’s goals. The role of self-control herein is to overcome these difficulties, thereby helping someone to achieve their goals. Therefore, the higher a person’ self-control, the more likely they are to achieve their goals, and the more likely they are then to experience a higher degree of subjective well-being. However, according to the energy model of self-control (Baumeister et al., 2007), the energy needed for employing self-control is limited, meaning that it can be exhausted and eventually lose its efficacy in the process of overcoming a given difficulty (this state of self-control is conceptualized by the term ego depletion); thus, individuals who deplete their energy herein would no longer be able to experience the benefits from goal achievement, implying that their subjective well-being would not improve. The question then arises, how can one maintain and supply the energy needed for self-control? If an individual is committed to achieving a desired goal by means of practicing self-control, then they would be motivated to maintain this limited source of energy. In this case, the role of motivation is critical, as it is defined as the inner strength that arouses, maintains, and promotes an individual’s activity toward achieving a certain goal. Motivation plays a role in the relationship between self-control and goal achievement and, subsequently, also influences the relationship between the latter and subjective well-being. Therefore, based on an individual’s goal, there are potential links between their self-control, motivation, and subjective well-being. For subjective well-being, goals often provide opportunities with which to experience more positive feelings (Joshanloo et al., 2020; Zeng and Chen, 2020). Additionally, pursuing goals exhausts the limited energy of self-control (Baumeister et al., 2007). Thus, by motivating individuals to pursue and maintain their goals would provide the extra mental strength needed to support their exhausted self-control (Muraven and Slessareva, 2003).

Some studies have provided evidence about the associations between these variables. Goal orientation usually involves two types of motivational orientations, namely the promotion motivation and prevention motivation (Higgins, 1997; Higgins et al., 2001; Scholer and Higgins, 2012). Although both types of motivation play a role in promoting a given behavior, they have different trade-off strategies between how they handle benefits and risks. Promotion motivation emphasizes the value of benefits, such as pursuing success, while prevention motivation focuses on the losses caused by risks, which includes actions around avoiding failure. Promotion motivation is concerned with nurturance needs, advancement-related goals, and eager approach strategies, while prevention motivation emphasizes safety and security needs, safety-related goals, and vigilant and avoidant strategies. Thus, from the perspective of motivation intensity, promotion motivation has a stronger power to promote goal seeking behaviors. Research has found that people with higher self-control were happier because of their more promotion-focused and less prevention-focused orientations (Cheung et al., 2014). Other studies have demonstrated that the interaction between state self-control (e.g., ego depletion) and motivation affects individual performance (Milyavskaya and Inzlicht, 2017). Furthermore, motivation and subjective well-being are also closely associated. In general, individuals with high motivation levels tend to experience a greater degree of happiness than those with low motivation levels (Li et al., 2015). Furthermore, considering the different types of motivation, individuals with a promotion orientation have reported higher subjective well-being scores than those with a prevention one (Manczak et al., 2014). This is because the former were more likely to be motivated to achieve their ideals and goals, which would then provide them with more opportunities to experience happiness. Therefore, based on the analyses of the relationship between self-control and subjective well-being, examining the effects of different motivation types would contribute to a more comprehensive understanding of this relationship (Ouyang et al., 2015; Zeng and Chen, 2020).

Muraven and Slessareva (2003) designed three experiments and found that motivation and self-control jointly determined individuals’ performance. Though they did not focus on subjective well-being, they identified the compensation effect of motivation on self-control. When in a state of ego depletion (which indicates that a person has less self-control energy), the participants with low motivation performed significantly poorly than those with high motivation. Based on this study, we speculated that there are several kinds of relationships between self-control and subjective well-being when considering the moderating role of motivation. When individuals have poor self-control abilities (e.g., they lack the self-control personality trait) or their self-control energy is insufficient (e.g., their state self-control is low), they can still be motivated to continuously pursue their goals if their motivation is strong (e.g., they possess a promotion-motivation orientation) or if they are aroused to a strong motivational state in a particular situation (e.g., when they adopt a state-specific promotion-motivation orientation; Muraven and Slessareva, 2003; Vohs et al., 2012; Cheung et al., 2014). As a result, they are still likely to achieve their goals and experience the positive outcomes caused by the fulfillment of these goals, thereby improving their subjective well-being (Joshanloo et al., 2020; Zeng and Chen, 2020). If they have a low level of motivation (e.g., they possess a prevention-motivation orientation) or if they are aroused to a weak motivational state in a particular situation (e.g., they experience a state-specific prevention-motivation orientation), they are more likely to give up on their goals when they experience a low sense of self-control. Hence, they would lose the opportunity to experience the resulting subjective well-being. When individuals experience a high level of self-control, notwithstanding the type of motivation, they are more likely to persist in pursuing their goals that primarily depend on their self-control, thus providing increased opportunities for improving their subjective well-being. Therefore, motivation would act as a moderator between self-control and subjective well-being. Among individuals with promotion motivation, there is no significant correlation between their self-control and subjective well-being because, whether their self-control is high or low, their subjective well-being remains high because of the compensatory role of prevention motivation. However, among individuals with a prevention motivation, there is a significant and positive correlation between self-control and subjective well-being. Because prevention motivation does not provide enough support for self-control, the pursuit of goals is completely dependent on the power of self-control itself. When self-control is strong, it is easier to achieve one’s goals, indicating that subjective well-being is high. When self-control is weak, however, it is difficult to achieve one’s goals, meaning that subjective well-being is low. Furthermore, according to regulatory focus theory (Higgins, 2005), a combination of both trait and state motivation has the best effect on achieving a goal (Lisjak et al., 2012); that is, an individual with a certain type of trait motivation could find themselves in a situation that then activates the corresponding type of state motivation. Therefore, the relationship between self-control and subjective well-being is most likely to be observed among individuals with prevention motivation in a situation that elicits state prevention motivation.

Although some studies have examined the relationship between motivation, self-control, and subjective well-being (Cheung et al., 2014; Ouyang et al., 2015; Nielsen et al., 2019), few have explained the uncertainty of the relationship between self-control and subjective well-being that include motivation as a moderator. Furthermore, studies have not produced consistent conclusions on the association between self-control and subjective well-being across different conditions, indicating that this relationship varies depending on different contexts. The moderator model is specifically used to explain how the link between two variables changes according to different levels of a given moderating variable. In the studies of Ouyang et al. (2015) and Nielsen et al. (2019), motivation was regarded as a mediating variable when examining the indirect relationship between self-control and subjective well-being (e.g., Cheung et al., 2014; Ouyang et al., 2015; Nielsen et al., 2019), but the uncertainty of this relationship could not be explained through the influence of motivation. The moderating-variable perspective identifies the complexity and various differential possibilities of the relationship between self-control and subjective well-being, which facilitates the integration of different findings in this field. Therefore, based on the moderating model of motivation, we explored the different relationships between self-control and subjective well-being. To analyze how this relationship changes under different circumstances, we focused on chronic personal traits and situationally induced states of self-control and motivation. These distinctions have been relatively under-scrutinized in studies. Hence, the role of self-control or motivation is understudied, indicated by the failure of extant literature to compare the effects of self-control levels in different states, lack of emphasis on the interaction of self-control and motivation on subjective well-being (e.g., Ouyang et al., 2015), and lack of examination into the classification of different types of motivation and self-control (e.g., Cheung et al., 2014; Nielsen et al., 2019).

In our research, the psychometrical model utilized focuses on the individual’s stable psychological traits, which can be used to understand trait self-control and their trait motivation orientation. Meanwhile, the experimental model utilized emphasizes each individual’s mental state in a certain context, wherein state self-control can be manipulated by altering the participants’ ego depletion, with state motivation then being elicited through incentives or priming. Studies have shown that the effect of self-control increased when the motivation levels of participants in the ego depletion condition were enhanced, while this effect decreased when their motivation was lowered (Vohs et al., 2012). This means that, when examining the effect of motivation as a moderator on the influence of self-control on subjective well-being, we need to not only consider trait self-control and trait motivation in terms of one’s personality but also include state self-control (e.g., that arising from ego depletion) and state motivation (e.g., that brought about by motivation priming) as well, which are triggered depending on the context. Some evidence has shown that trait motivation’s effects do differ depending on a person’s individual characteristics—with state motivation being triggered by situational factors—in terms of their self-control and happiness (Ouyang et al., 2015).

To date, most studies have been based on a psychometric model that analyzed the association between self-control and subjective well-being while focusing on trait self-control and rarely examining state self-control. Ego depletion is a typical indicator of state self-control, but its role has not been fully explored in this line of research. In the limited literature, all participants experienced the same levels of ego depletion. Furthermore, there was a lack of comparative data from the non-depletion group or, in some cases, only the conditions between depletion and non-depletion were compared while the degree of ego depletion was ignored. Muraven and Slessareva (2003) found that motivation influenced the self-control of depleted participants but had no effect on non-depleted ones. In a set of sequential self-control tasks, however, individual performance was found to vary with one’s degree of ego depletion; specifically, individuals’ self-control worsens when they are already heavily depleted when confronted with subsequent depletion tasks (Baumeister et al., 2007). This suggests that mild and severe ego depletion may have differential effects. Research has found that increasing motivation only assuages the adverse effects of mild ego depletion, but does not affect severe depletion (Vohs et al., 2012). Additionally, scholars have suggested that there exists a curvilinear relationship between self-control and happiness, arguing that having either too high or too low self-control may have a negative effect on happiness (Carter et al., 2015). In contrast, other scholars have denied this view (Wiese et al., 2018). All these results imply that it is necessary to comprehensively consider different levels of ego depletion when manipulating state self-control (i.e., not only dividing the conditions into depletion and non-depletion ones but also distinguishing between mild and severe depletion).

In summary, the present study explored the moderating role of motivation in the relationship between self-control and subjective well-being with respect to both trait and state motivation. Specifically, Study 1 examined the moderating role of trait motivation (comparing promotion vs. prevention orientations) in the relationship between trait self-control and subjective well-being. We hypothesized that a positive influence of trait self-control on subjective well-being would be observed among individuals with a prevention-motivation orientation. Study 2 examined the subjective well-being of individuals with two types of trait motivations under different ego depletion conditions (no depletion vs. mild depletion vs. severe depletion) when priming two types of state motivations (promotion vs. prevention). We hypothesized that the positive relationship between state self-control and subjective well-being would only appear in the case of state or trait prevention motivation, with this relationship being most significant when the motivation types are congruent.

## Study 1

### Materials and Methods

#### Participants

*A priori* power analysis was conducted using G^–^Power (version 3.1, Faul et al., 2009) to determine the minimum required sample size. This analysis revealed that a sample size of 134 would have sufficient power to detect a medium effect size (0.3), with an α level of 0.05, and a power (1-β) of 95%. Participants were recruited from three universities located in Northwestern China. After signing an informed consent form, 400 undergraduates participated in the survey, with 352 eventually completing all of the study questionnaires, resulting in a response rate of 88%. Notably, the final sample size met the statistical conditions. The participants were from both urban and rural areas and included Han, Hui, Tibetan, and other major ethnic minorities living in the Northwestern region. The final sample consisted of 352 Chinese undergraduate students of ages 18–23 years (*M*_age_ = 20.02, *SD*_age_ = 1.13; 255 females).

#### Measures

##### Trait Self-Control

Self-Control and Self-Management Scale (SCMS; Mezo, 2005) was used to measure respondents’ trait self-control. The Chinese version of the SCMS (Zhao, 2016) consists of 16 items that are rated on a five-point scale (1 = not at all, 5 = very much). Five items are reversed so that a higher total score indicated a higher level of trait self-control. The reliability of the scale was good, with a Cronbach’s α of 0.87.

##### Subjective Well-Being

Subjective well-being was evaluated using two dimensions: life satisfaction and affect experience. Life satisfaction was measured using the Chinese version of the five-item Life Satisfaction Rating Scale (LSRS; Diener et al., 1985), with an example item being: “I am satisfied with my life.” In this scale, participants rated each item on a five-point scale (1 = strongly disagree, 5 = strongly agree). Higher scores represent higher levels of life satisfaction. The reliability of the scale was good, with a Cronbach’s α of 0.84. Meanwhile, the Chinese version (Qiu et al., 2008) of the Positive Affect and Negative Affect Scale (PANAS, Watson et al., 1988) was used to measure respondents’ affect experiences. The PANAS is an 18-item scale that contains two subscales: positive affect (e.g., being active, enthusiastic, and excited) and negative affect (e.g., being afraid, scared, and nervous). Participants rate the items on a five-point scale (1 = strongly disagree, 5 = strongly agree). The reliabilities of these two subscales were good, with a Cronbach’s α of 0.86 and 0.81, respectively. After the negative affect items are reversed, a higher total score on both scales indicates higher subjective well-being.

##### Trait Motivation

The Chinese version (Ye, 1992) of the Achievement Motivation Scale was used to measure respondents’ trait motivation using a five-point scale (1 = strongly disagree, 5 = strongly agree). The scale contains 15 items that comprise two dimensions. The first dimension is the respondent’s motive to achieve success (e.g., “I feel pleasure when working on tasks that are somewhat difficult for me”), which belongs to the promotion-motivation orientation and evaluates an individual’s willingness to risk success, with higher scores indicating a higher promotion motivation. The second dimension is the respondent’s motive to avoid failure (e.g., “I become anxious when I encounter a problem I don’t understand at once”), which belongs to the prevention-motivation orientation and evaluates an individual’s willingness to reduce risk and losses, with higher scores indicating a higher prevention motivation. The reliabilities of these two subscales were good, with Cronbach’s αs of 0.87 and 0.86, respectively.

#### Procedure

All participants completed the questionnaires anonymously and were informed that their participation in this study was voluntary, that they were free to withdraw at any time, and that the data were only going to be used for research purposes. As a reward, each participant received a gift worth 10 yuan at the end of the survey. The testing materials and survey procedures were approved by the Ethics in Human Research Committee of the School of Psychology, Northwest Normal University.

#### Statistical Analysis

The first step undertaken was to perform a descriptive analysis of the variables (means and standard deviations). The relationships between trait self-control, subjective well-being, and trait motivation were examined using Pearson’s correlation. We also explored the correlation between age and these variables using the same method, with gender differences between these variables being examined using an independent samples *t*-test. Next, to test whether trait motivation moderates the association between trait self-control and subjective well-being, the moderation model was tested using Model 1 of the PROCESS 3.3. by Andrew F. Hayes for SPSS 24. In particular, one of two types of motivation (i.e., either promotion or prevention motivation) was set as the moderator variable, with trait self-control being set as the independent variable (X variable), and subjective well-being as the dependent variable (Y variable). Age and gender (0 = male, 1 = female) were used as the control variables (covariates). Furthermore, simple slope analyses were used to explore the relationship between trait self-control and trait prevention motivation in the low and high motivation groups.

### Results

#### Common Method Biases

In the present study, a common method bias may have occurred because all data were derived using self-report measures. Thus, prior to the data analysis, a Harman’s one-factor test was conducted, wherein 18 factors with eigenvalues above one were extracted. The results indicated that the first factor explained 13.76% of the variance, which was much lower than the critical value of 40%. Therefore, there was no serious concern around a common method bias occurring in this study.

#### Descriptive Statistics and Correlation Analysis

Descriptive statistics were first determined for trait self-control, subjective well-being, and trait motivation. A Pearson’s correlation analysis was performed to examine the relationships between these variables (Table 1). The results revealed that trait self-control was positively correlated with subjective well-being and that the relationships between the two types of trait motivation and other variables varied. Trait promotion motivation was positively correlated with trait self-control and subjective well-being, while trait prevention motivation was negatively correlated with trait self-control and was not correlated with subjective well-being. Moreover, trait promotion motivation was negatively correlated with trait prevention motivation. Additionally, we explored the potential effects of two demographic variables, gender and age, which have been used as controlled variables in previous studies (Diener et al., 1999; Chen, 2013). The results of the Pearson’s correlation analysis revealed that there was no significant correlation between age and self-control or between the two types of trait motivation and subjective well-being. An independent samples *t*-test was used to examine the gender differences in these variables. The results outlined that there were no significant differences herein, except for self-control (*M*_male_ ± *SD*_male_ = 3.42 ± 0.46, *M*_male_ ± *SD*_female_ = 3.62 ± 0.44, *t* = –3.334, *p* = 0.001).

**TABLE 1:** Descriptive statistics and correlations among variables (*N* = 352).

	*M*	*SD*	1	2	3
1 Trait self-control	3.59	0.45	–		
2 Trait promotion motivation	3.11	0.42	0.367***	–	
3 Trait prevention motivation	3.01	0.48	–0.147**	–0.220***	–
Subjective well-being	2.43	0.44	0.336***	0.335***	0.027

**p < 0.01; ***p < 0.001.

#### Moderator Analyses

**FIGURE 1:**
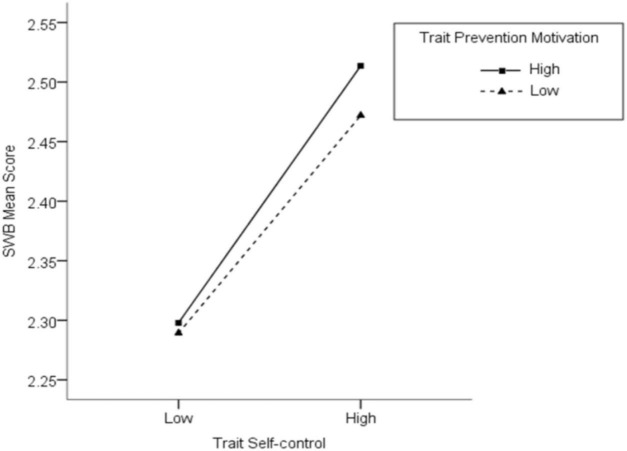
Interaction between trait self-control and trait prevention motivation on subjective well-being.

After controlling for the potential effects of age and gender, the moderating models of the two types of motivation were examined separately. Regarding the moderating model of trait prevention motivation, trait self-control significantly predicted changes in subjective well-being; however, trait prevention motivation did not. More importantly, the interaction between trait prevention motivation and trait self-control, according to a bootstrap confidence interval (95% CI) that did not include 0, identified a significant effect herein. This indicated that trait prevention motivation was a significant moderator of the relationship between trait self-control and subjective well-being (Table 2). A simple slope analysis was also performed to further explore the moderating mechanism of trait prevention motivation. The scores of trait prevention motivation were subtracted from two specific points (M + SD and M – SD) to determine the high and low levels. Simple slope analyses indicated that trait self-control was significantly associated with subjective well-being at both high (*simple*_slope_ = 0.507, *t* = 6.967, *p* < 0.001) and low levels (*simple*_slope_ = 0.217, *t* = 3.266, *p* = 0.001) of trait prevention motivation, but that its predictive strength weakened when the level of prevention motivation was decreased. According to the trend of Figure 1, which included both low and high levels of prevention motivation, the relationship between self-control and subjective well-being demonstrated the same trend. Specifically, the respondents’ level of subjective well-being increased with their level of self-control. However, the strength of this change was different. For those with a high prevention motivation, this positive relationship was more obvious.

**TABLE 2:** Moderating analysis of trait prevention motivation on the relationship between trait self-control and subjective well-being.

	β	*t*	95%CI	*ΔR* ^2^	*F*
A: Trait self-control	0.362	6.918***	[0.249, 0.484]		
B: Trait prevention motivation	0.071	1.486	[–0.035, 0.167]		
A × B	0.302	3.151**	[0.089, 0.504]	0.026	9.929**
Gender	0.021	0.351	[–0.099, 0.135]		
Age	0.013	0.631	[–0.026, 0.051]		

**p < 0.01, ***p < 0.001.

Regarding the moderating model of trait promotion motivation, both it and trait self-control significantly predicted subjective well-being. However, the interaction between these two variables in a bootstrap confidence interval did not include 0, indicating that trait promotion motivation was not a significant moderator (Table 3).

**TABLE 3:** Moderating analysis of trait promotion motivation on the relationship between trait self-control and subjective well-being.

	β	*t*	95%CI	Δ*R*^2^	*F*
A: Trait self-control	0.251	4.614***	[0.125, 0.392]		
B: Trait promotion motivation	0.250	4.496***	[0.123, 0.385]		
A × B	0.002	0.024	[–0.207, 0.293]	0.000	0.001
Gender	0.053	0.905	[–0.069, 0.175]		
Age	0.018	0.891	[–0.022, 0.055]		

***p < 0.001.

### Discussion

In its examination of personality traits, Study 1 explored the moderating role of trait motivation on the relationship between trait self-control and subjective well-being using a psychometric model. The results showed that generally, the higher the level of trait self-control, the higher a person’s overall subjective well-being. However, this relationship is moderated by the prevention motivation; specifically, the positive influence of self-control on subjective well-being becomes more obvious when the level of trait prevention motivation increases. This finding supports our hypothesis. We speculated that, among individuals with prevention motivation, there is a significantly positive correlation between self-control and subjective well-being. Because prevention motivation does not provide enough support for a lack of self-control, it becomes difficult to achieve one’s goals, as well as maintain a low level of subjective well-being, when self-control is weak. In contrast, it is easy to achieve goals and subjective well-being is high when self-control is strong.

Although the association between trait self-control and subjective well-being has been controversial, most researchers have argued that there is a positive link between the two (Cheung et al., 2014; Grund et al., 2015; Wiese et al., 2018; Nielsen et al., 2019; Massar et al., 2020). Study 1 supports this view and further finds that the association is moderated by the trait prevention-motivation orientation. Prevention motivation falls under the theme of avoidance motivation, wherein individuals attempt to reduce risk and losses in pursuit of security goals (Higgins, 1997). Researchers argue that the positive link between self-control and subjective well-being is attributable to the function of self-control in assisting individuals in achieving their goals, as it creates more opportunities to experience positive life satisfaction and affect, while avoiding failure-induced negative affect and stress (Hofmann et al., 2014; De Ridder and Gillebaart, 2017). Self-control is not only an attempt to restrain one’s temptations (Baumeister et al., 1998), but also involves the adoption of various strategies in order to achieve one’s desired goals (Nielsen et al., 2019); its function is similar to that of promotion motivation (Cheung et al., 2014; Gillebaart et al., 2016). Therefore, the effect of self-control on subjective well-being is less obvious in individuals with trait promotion motivation because it has a similar positive relationship with happiness as does self-control (Manczak et al., 2014; Ouyang et al., 2015). Some studies have provided evidence that people with high self-control do not necessarily experience more happiness because their motivation orientation influences this relationship; in other words, individuals with trait promotion motivation experience greater levels of happiness regardless of their own self-control abilities (Ouyang et al., 2015). Conversely, for individuals with prevention motivation, the effect of self-control on their subjective well-being is more obvious because this motivation orientation encourages them to adopt more conservative strategies, which often results in the behavioral intention of them maintaining the status quo instead of taking risks to achieve one’s goals (Higgins, 1997; Scholer and Higgins, 2012). Prevention motivation is not likely to encourage individuals to explore new opportunities, which may be beneficial to one’s subjective well-being. Thus, prevention motivation is not as closely related to happiness as is promotion motivation (Cheung et al., 2014). This means that individuals with prevention motivation achieve their desired goals only through the power of self-control, consequently having more positive experiences. In these cases, the role of self-control is fully displayed. As a result, the positive connection between self-control and subjective well-being is more significant among individuals with prevention motivation. Our hypothesis was confirmed through the findings of Study 1.

To the best of our knowledge, few studies have focused on the influence of trait motivation on the relationship between self-control and subjective well-being. One study found that motivation plays a mediating role between trait self-control and subjective well-being (Cheung et al., 2014). Specifically, it found that trait self-control has a positive relationship with the promotion motivation, which is positively associated with happiness. It also found that trait self-control has a negative relationship with the prevention motivation, which is negatively associated with happiness. The aforementioned study’s research question was different from that of the present one because we emphasized motivation as a moderator instead of as a mediator. Nonetheless, both studies would help scholars to comprehensively understand the mechanism of self-control and its influence on subjective well-being. In addition to stable trait self-control and trait motivation, situational factors also need to be considered, including conditions that trigger state self-control (e.g., ego depletion) and state motivation. Therefore, in Study 2, we explored the moderating effect of trait and state motivation on the relationship between state self-control and subjective well-being in an experimental model.

## Study 2

### Materials and Methods

#### Participants and Design

Study 2 utilized a 2 (trait motivation: trait promotion vs. trait prevention motivation) × 2 (state motivation: state promotion vs. state prevention motivation) × 3 (ego depletion: no depletion vs. mild depletion vs. severe depletion) three-factor mixed experiment design, wherein both trait and state motivation were used as the between-group variables and ego depletion was used as the within-group variable. In this design, ego depletion was the independent variable that represented the respondent’s state of self-control, with subjective well-being being the dependent variable. Finally, both trait and state motivation were set as the moderating variables.

G^–^Power (version 3.1, Faul et al., 2009) revealed that a sample size of 124 was required for a power (1-β) of 95% to detect an effect of *F* = 0.25 at α = 0.05. Based on the participants’ scores in the Achievement Motivation Scale in Study 1, those with typical trait promotion or prevention motivation were selected by adding or subtracting one standard deviation from the mean. One month after Study 1, we contacted the participants who met our selection criteria, with 160 then volunteering to take part in Study 2. After removing all invalid data, we obtained valid responses from 138 participants (*M*_age_ = 20.24 years, *SD*_age_ = 1.34; 97 females), including 64 with trait promotion motivation and 74 participants with trait prevention motivation.

#### Materials

##### Motivation Priming Materials

State motivation is triggered through the process of motivational priming. To ensure the validity of the priming manipulation method used, we conducted a three-step work. First, we identified the definitions of promotion and prevention motivation. Stories illustrating promotion motivation emphasize the value of benefits, such as pursuing success; the characters in these stories would be described as individuals who adopt eager approach strategies, are willing to take risks, and are eager to succeed for nurturance needs. Meanwhile, prevention motivation focuses on the losses caused by risks, such as avoiding failure. Stories illustrating this motivation would have characters who adopt vigilant and avoidant strategies and who are more willing to pass up tempting but risky opportunities for safety reasons. Second, six stories were selected from mass media based on the definitions of these two motivation types, with three stories related to promotion motivation and another three related to prevention motivation. We made the appropriate adjustment on the format and word count of each one to make them as similar as possible in both reading time and difficulty. Additionally, there were no obscure or difficult words used in any of these stories. The average reading time for each one was about 2 min. Third, from a personality psychology class, we enlisted 30 graduate students (18 female) to evaluate the materials on motivation. These graduate students were majoring in psychology and their mean age was 22.31 years. They were asked to evaluate the validity of the experimental materials. They rated the six stories on a five-point scale based on the definitions of the two types of motivation orientation. After reading each story, they were asked to answer the following question: “Do you think this story is a good example of this type of motivation?” (1 = not at all, 5 = very much). Finally, we selected the most representative story for each motivation type as the priming material (*M*_promotion_ = 4.12, *SD*_promotion_ = 0.33; *M*_prevention_ = 4.46, *SD*_prevention_ = 0.50).

##### Ego Depletion Procedure

**FIGURE 2:**
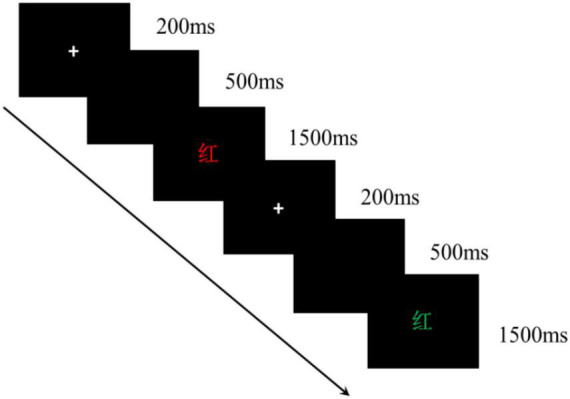
The procedure of Stroop task. The Chinese character of “红” means “red.”

Ego depletion was assessed using the Stroop task, with the target stimuli being Chinese characters with red, yellow, green, and blue color words. The participants were asked to make a fast and accurate judgment of the target stimulus’ color by pressing different keys when it was presented, with the colors “red,” “yellow,” “green,” and “blue” corresponding to the “D,” “F,” “J,” and “K” keys, respectively. The practice phase was set up for 32 trials to ensure that the participants were familiar with the experimental procedure. During the actual formal stage, there were 80 trials. The specific process is shown in Figure 2. The gaze point shown by the “+” symbol was first presented, followed by a blank screen. Then, a Chinese character stimulus was presented. If the participants responded during this period, the stimulus disappeared and the next trial proceeded. If they did not respond, the stimulus disappeared automatically, implying that the trial was invalid, and the next trial followed. For the no depletion task, the word’s color was consistent with the word’s meaning; for the mild depletion task, the word’s color was inconsistent with the word’s meaning; and for the severe depletion task, the interference of auditory stimuli was added to the inconsistent visual stimuli (i.e., inconsistent word’s colors and meanings). For example, when the participants gazed at the stimulus, they heard the sound of a word’s meaning via headphones, with the audio duration being the same as that of the visual stimulus. Thus, when the participants saw the word “blue” in a red font and simultaneously heard the word “blue,” the correct response was “red” (i.e., correctly answered by pressing the “D” key). The experimental procedure was developed using E-Prime 2.0, with a total of 112 trials per experiment condition. Subjective well-being was measured using the same questionnaires as used in Study 1.

#### Procedure

The participants completed all experimental tasks on three consecutive Friday afternoons. Upon arriving at the laboratory at the designated time on the first Friday, the participants completed the no depletion task and, then, reported their level of fatigue on a seven-point scale (1 = not fatigued, 7 = very fatigued). Half of the participants for each type of trait motivation were then assigned to the promotion-motivation priming condition, while the other half were assigned to the prevention-motivation priming condition. After reading the story about the given motivation orientation, the participants’ temporary risk propensity was tested on a seven-point scale (“Are you willing to take a risk in pursuit of success now?” 1 = very unwilling, 7 = very willing). Finally, all participants completed questionnaires on their subjective well-being. The mild depletion task was completed on the second Friday, and the severe depletion task, on the third Friday. The experimental procedures in all three sessions were identical. According to the experimental design, there should have been 40 participants divided into each type of state motivation in the priming condition (Table 4); however, 12 participants did not participate in the subsequent experiments, six failed the Stroop task, and four did not complete at least one of the subjective well-being questionnaires. Thus, the number of valid responses came to 138. Each participant also received a small token of appreciation.

**TABLE 4:** Descriptive statistics of participants’ subjective well-being in each condition.

Ego depletion	Trait motivation	State motivation	*N*	*M*	*SD*
No depletion	Promotion	Promotion	36	3.51	0.37
		Prevention	28	3.58	0.37
	Prevention	Promotion	40	3.23	0.28
		Prevention	34	3.26	0.49
Mild depletion	Promotion	Promotion	36	3.49	0.30
		Prevention	28	3.57	0.50
	Prevention	Promotion	38	3.14	0.31
		Prevention	34	3.06	0.50
Severe depletion	Promotion	Promotion	36	3.49	0.30
		Prevention	28	3.52	0.42
	Prevention	Promotion	38	3.08	0.31
		Prevention	34	3.22	0.47

### Results

Descriptive statistics were used to calculate the means and standard deviations of each condition. The validity of the experimental manipulation of ego depletion was then tested using an independent samples *t*-test. Furthermore, the main effects and interactions of ego depletion, trait motivation, and state motivation on respondents’ subjective well-being were explored using a repeated measures analysis of variance (ANOVA). If the interaction between an independent (ego depletion) and a moderating variable (state motivation or trait motivation) is significant, it would prove that motivation has a moderating effect on the relationship between state self-control and subjective well-being. Following this, a simple effect analysis was used to explore the effects of state self-control on subjective well-being in different conditions of state or trait motivation.

#### Manipulation Check

First, an independent samples *t*-test was conducted to evaluate the validity of the experimental manipulation. For ego depletion, the feeling of fatigue differed across the various ego depletion conditions, with participants reporting significantly less fatigue in the no depletion condition (*M* = 3.64, *SD* = 1.34) than in the mild depletion [*M* = 6.09, *SD* = 1.15; *t*(137) = –15.31, *p* < 0.001, *d* = 1.96] and the severe depletion conditions [*M* = 6.50, *SD* = 1.12; *t*(137) = –16.69, *p* < 0.001, *d* = 2.32]. The feeling of fatigue in the mild depletion condition was also significantly less than that in the severe depletion condition [*t*(137) = –3.486, *p* = 0.001, *d* = 0.36], indicating that our manipulation of ego depletion was valid. For state motivation, the risk propensity of the participants in the promotion-priming condition (*M* = 6.21, *SD* = 1.62) was significantly higher than that of those in the prevention-priming condition [*M* = 4.44, *SD* = 1.40; *t*(136) = 6.806, *p* < 0.001, *d* = 1.17], indicating that motivation priming effectively initiated the two state motivation orientations.

#### Descriptive Statistics of Subjective Well-Being in Each Condition

Descriptive statistics were used to calculate the means and standard deviations of each condition (Table 4). Table 4 shows the participants’ subjective well-being scores for each type of state motivation in the priming condition wherein they accomplished different ego depletion tasks with different trait motivation orientations.

#### Main Effect of and Interaction of Self-Control and Motivation

A repeated measures ANOVA revealed the existence of a significant main effect of trait motivation [*F*(1, 136) = 22.00, *p* < 0.001, η^2^ = 0.142], a critically significant main effect of ego depletion [*F*(2, 274) = 2.434, *p* = 0.090, η^2^ = 0.018], and a non-significant main effect of state motivation [*F*(1, 136) = 0.55, *p* = 0.461]. *Post hoc* multiple comparisons revealed that individuals with trait promotion motivation had significantly higher levels of subjective well-being than those with trait prevention motivation (*p* < 0.05). In terms of second-order interactions, the interaction between ego depletion and trait motivation was significant [*F*(2, 274) = 6.848, *p* = 0.001, η^2^ = 0.049]; the interactions between ego depletion and state motivation [*F*(2, 274) = 2.265, *p* = 0.106], and between trait and state motivation [*F*(1, 134) = 0.149, *p* = 0.700] were not significant. In terms of third-order interactions, the interaction between ego depletion, state motivation, and trait motivation was significant, [*F*(2, 268) = 5.586, *p* = 0.004, η^2^ = 0.040]. These findings indicate that trait motivation moderated the relationship between state self-control and subjective well-being, but state motivation did not. However, state motivation demonstrated a moderating effect when combined with trait motivation.

**FIGURE 3:**
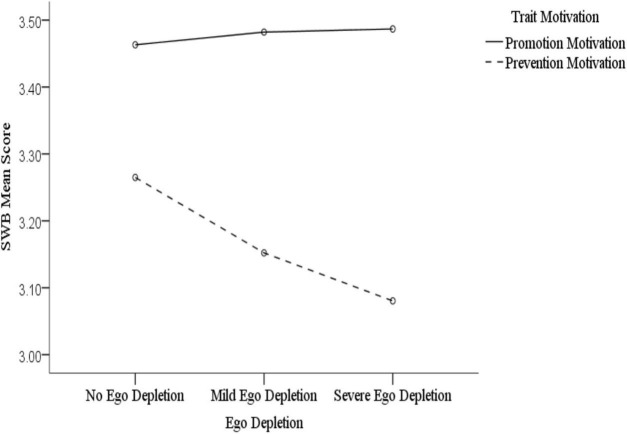
The effect of ego depletion on subjective well-being in the level of state promotion motivation.

**FIGURE 4:**
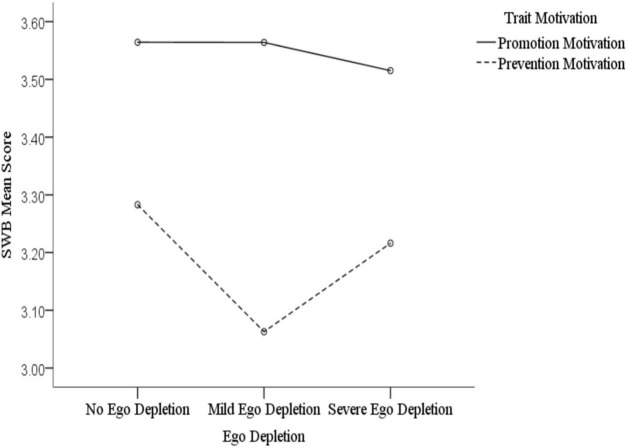
The effect of ego depletion on subjective well-being in the level of state prevention motivation.

Figures 3, 4 show that, across different levels of state motivation, individuals with trait promotion motivation reported different levels of subjective well-being compared to those with trait prevention motivation under different ego depletion conditions. Specifically, our simple effect analysis revealed that, according to the level of state promotion motivation, the subjective well-being of individuals with trait promotion motivation did not significantly differ after they had completed different depletion tasks; however, there was a significant difference observed between the no depletion and severe depletion conditions among individuals with trait prevention motivation (*t* = 8.958, *p* < 0.001, *d* = 0.51). Figure 3 shows a gradual decline in respondents’ subjective well-being as their ego depletion became more severe. This indicates that there is a positive relationship between state self-control and subjective well-being under the condition of experiencing a combination of state promotion motivation and trait prevention motivation. According to the level of state prevention motivation, the subjective well-being of individuals with trait promotion motivation differed significantly only between the no depletion and severe depletion conditions (*t* = 2.105, *p* = 0.045, *d* = 0.15), which showed a gradual decline of subjective well-being with ego depletion (Figure 4), indicating that the positive relationship between state self-control and subjective well-being exists in the condition involving the combination of state prevention motivation and trait promotion motivation. In addition, there were significant differences observed in all three ego depletion conditions among individuals with trait prevention motivation. Specifically, there were significant differences found between the no depletion and mild depletion conditions (*t* = 6.901, *p* < 0.001, *d* = 0.40), the no depletion and severe depletion conditions (*t* = 3.788, *p* = 0.001, *d* = 0.08), and the mild depletion and severe depletion conditions (*t* = –3.855, *p* = 0.001, *d* = 0.33). This means that self-control and happiness do not possess a simple linear relationship under the condition of a combination of state and trait prevention motivation. There was a positive correlation between them from no to mild ego depletion, with a negative correlation from mild to severe ego depletion. Overall, these results indicate that the positive relationship between state self-control and subjective well-being does not always exist and depends on the type and nature of the individual’s motivation. In most cases, there is a positive association between the two; however, a negative one does occur under the combined condition of trait and state prevention motivation.

### Discussion

Study 2 explored the relationship between state self-control (ego depletion), state motivation, and subjective well-being from a contextual perspective in an experimental model; that is, it considered the effects of situational factors on subjective well-being in addition to stable personality factors. The results revealed that the experimental manipulations of the motivation priming and ego depletion were effective, indicating that state self-control and state motivation are easily induced in certain contexts. This finding suggests that it is worthwhile to explore features that change with one’s surroundings, as opposed to only focusing on a person’s stable or constant personality traits. Our findings are consistent with those of previous studies, wherein some scholars have identified traits and states that should be distinguished when examining the roles of self-control (Cunningham and Baumeister, 2016; Friese et al., 2018) or motivation (Silva et al., 2014; Vanroy et al., 2019).

By fully considering both trait and state motivation, we found that these two factors interacted significantly with state self-control (i.e., ego depletion), which indicates that personal and situational factors jointly influence subjective well-being. Study 2 therefore supports our hypothesis; that is, the positive relationship between state self-control and subjective well-being only appears among people with either state or trait prevention motivation, with this relationship being most significant when these two motivation types are congruent. We found that the positive relationship between state self-control and subjective well-being exists under the conditions of a combination of state promotion and trait prevention motivation and a combination of state prevention motivation and trait promotion motivation. Furthermore, under the condition of a combination of state and trait prevention motivation, there exist both positive and negative associations between state self-control and subjective well-being. In the case when self-control is compensated by adopting a promotion motivation and not a prevention one, there is no significant relationship between state self-control and subjective well-being when state and trait promotion motivations are combined. This is because promotion motivation can inspire individuals to pursue goals despite their lowered self-control, which means that their subjective well-being is not accompanied by changes in self-control. Additionally, in other combinations that include prevention motivation, a positive association can be observed. However, we are unsure as to why subjective well-being increased in the range between mild and severe ego depletion in the matching condition of state and trait prevention motivation. This is a question that needs to be explored by future researchers.

On the one hand, the moderating effect of trait prevention motivation on the relationship between self-control and subjective well-being was significantly stronger than that of trait promotion motivation. On the other hand, the relationship between self-control and subjective well-being was more obvious in the state prevention-motivation condition than in the state promotion-motivation one. These results correspond to those of Study 1. Similar to the assumption in Study 1, we speculated that individuals with prevention motivation are less motivated and require greater self-control abilities to achieve long-term goals and create more opportunities to experience happiness; meanwhile, individuals with promotion motivation can compensate for the shortage of strength caused by ego depletion to some extent because they are better able to maintain their behavior in these cases (Weinberg and Gould, 2015). Therefore, promotion motivation can maintain the effect of self-control on subjective well-being, but prevention motivation cannot, as it results in a diminished effect of self-control on subjective well-being after energy depletion. According to the regulatory focus theory (Higgins, 2005), the effect of motivation is maximized when one’s trait matches their current state. Our study findings were in line with the theory, in that trait prevention motivation had the most significant effect on the link between ego depletion and subjective well-being in the state prevention-motivation condition.

Study 2 found that, although ego depletion reduced participants’ subjective well-being levels, this relationship’s trend was not exactly linear. While in the state promotion-motivation condition, there was no significant difference observed between no depletion and mild depletion, or between mild depletion and severe depletion. In the state prevention-motivation condition, there was a lower level of subjective well-being in the mild depletion than in the no depletion and severe depletion conditions. One previous study found that strong motivation priming mitigated mild ego depletion but had no effect on severe depletion (Vohs et al., 2012). Other studies have suggested that the relationship between self-control and subjective well-being may be curvilinear; arguing that having a too high or a too low level of self-control may have detrimental effects on happiness (Carter et al., 2015; Wiese et al., 2018). Although the results of this study are different from these prior ones, they still illustrate that the degree of ego depletion must be comprehensively considered when evaluating state self-control.

There are some studies that echo ours. For example, Shah et al. (1998) found that individuals with a prevention motivation perform well in “avoid a loss” situations; however, they did not examine the role of self-control in these contexts. Other studies have explored the interaction between ego depletion and motivation (Berkman et al., 2017), but they did not link this effect to subjective well-being. From an experimental perspective, Study 2 further demonstrated that motivation plays a moderating role in the relationship between self-control and subjective well-being, including both trait motivation (representing personality factors) and state motivation (representing contextual factors).

## General Discussion

The present research verified the moderating role of motivation in the relationship between self-control and subjective well-being, especially among participants with a prevention-motivation orientation. According to the literature, there is a positive association between self-control and subjective well-being; that is, subjective well-being tends to increase with a higher level of self-control (Cheung et al., 2014; Grund et al., 2015; Wiese et al., 2018; Nielsen et al., 2019; Massar et al., 2020). This can be explained by the nature of self-control, which involves the overriding or inhibiting of automatic, habitual, or innate behaviors, urges, emotions, or desires that would otherwise interfere with a person’s goal-directed behaviors (Baumeister et al., 1994; Barkley, 1997). Our research supports this conclusion. More importantly, we verified the moderating role of motivation in this relationship. We assumed that the impact of self-control on subjective well-being changes depending on the type of motivation.

Self-control aims to restrict a person’s adverse impulsive behaviors in order to make it easier for them to achieve their rational goals, which could grant people access to success, which, in turn, provides them with more opportunities to achieve greater life satisfaction and positive affect. Therefore, there is a positive relationship between self-control and subjective well-being. However, self-control is challenged by the energy loss that occurs in the process of pursuing one’s goals. In this case, if an individual has a strong desire to achieve a given goal, such as through adopting promotion motivation, any deficit in their self-control will be compensated. Because promotion motivation focuses on the value of success, it inspires individuals to take more risks in order to achieve success; therefore, individuals with this type of motivation have a more powerful drive to overcome difficulties, which is beneficial in terms of maintaining a degree of self-control. It ensures a high level of happiness even at a lower level of self-control. However, when a person has sufficient self-control energy, which is not sensitive to their type of motivation (Muraven and Slessareva, 2003), they are more likely to achieve their goals by using their self-control’s innate strength, thus creating greater opportunities to increase their happiness. This means that it is possible to achieve one’s goals no matter how much their self-control energy changes, and thus, individuals’ happiness levels are more likely to remain high. However, a significant correlation supporting this was not observed. On the contrary, if individuals have a weak desire to achieve their goals, such as those who adopt a prevention-motivation orientation, their self-control will not benefit from this motivation orientation. Because prevention motivation focuses on the negative effects of failure, encouraging people to adopt more conservative strategies in order to avoid failure, it does not provide sufficient motivation to solve any problems that arise subsequently; hence, their subjective well-being cannot be improved because their goals are less likely to be achieved. The relationship between subjective well-being and self-control is not affected by prevention motivation, with subjective well-being maintaining its positive links with self-control herein. Therefore, motivation type plays a moderating role in the association between self-control and subjective well-being.

Although a few studies have discussed the relationship among these three variables (Cheung et al., 2014; Ouyang et al., 2015; Nielsen et al., 2019), there are still some gaps in the literature, including the fact that the moderating role of motivation has not been analyzed, the interaction effect between personal and situational factors has not been explored, and multiple levels of ego depletion have never been fully manipulated. Considering the above research gaps, the present study examined the moderating effects of motivation on the relationship between self-control and subjective well-being. In both our psychometrical and experimental models, we found that motivation has a moderating effect, with this effect being more pronounced in the case of prevention motivation. According to the results of the two studies, the moderating effect of trait motivation is always present, while that of state motivation needs to occur in conjunction with trait motivation in order to exert any influence. This suggests that distinguishing between trait and state motivations is necessary for a comprehensive understanding of the mechanism underlying a person’s motivation.

The present findings echo those of previous studies, meaning that it is necessary to further explore the link between self-control and subjective well-being from the perspective of motivation (Cheung et al., 2014; Ouyang et al., 2015; Nielsen et al., 2019; Zeng and Chen, 2020). Additionally, our findings also address various issues ignored in the literature. When considering the roles of the two types of motivation, studies have tended toward supporting the belief that promotion motivation has a more positive effect (Ouyang et al., 2015; Weinberg and Gould, 2015); however, prior research has also found that the effects of prevention motivation are more obvious when self-control resources are inefficient (Lisjak and Lee, 2014). The present study draws the same conclusion. Additionally, we found that, when an individual had a state prevention-motivation orientation, their subjective well-being was lower in the mild depletion condition than in the no depletion and severe depletion ones. This finding contrasts with the beliefs of other researchers who posited that too high or too low levels of self-control may interfere with a person’s happiness (Letzring et al., 2005; Tay and Diener, 2011). There is evidence that contradicts this line of reasoning (Wiese et al., 2018). Hence, this issue needs to be further discussed and explored in future research. Overall, our results support our hypothesis that motivation moderates the relationship between self-control and subjective well-being. Given that both promotion motivation and self-control adopt the goal-directed functions of maintaining and promoting target-oriented behaviors, the effect of self-control on subjective well-being is not as obvious among individuals with this kind of motivation. As for individuals with prevention motivation, the incentivizing role of self-control can compensate for the weaknesses associated with this motivation orientation, and thus, it demonstrates a more obvious effect.

Although the present study explored the relationship between self-control, subjective well-being, and motivation from various aspects, it had several limitations. First, we only focused on subjective well-being—rather than psychological well-being—when discussing happiness. Research has demonstrated that self-control and psychological well-being are related, with self-control being able to enhance a person’s psychological well-being (Fritz and Gallagher, 2019). Some researchers have also argued that the link between psychological well-being and the variables involved in pursuing future objectives (e.g., self-control) is stronger than the link between subjective well-being and these variables (Joshanloo et al., 2020). Because psychological well-being emphasizes the importance of achieving one’s values, it would have some connection to a persons’ motivation and their degree of self-control. Subjective well-being may also be referred to as “hedonic well-being,” which is usually evaluated through one’s affect and life satisfaction (Diener et al., 2018); meanwhile, psychological well-being can be referred to as “eudaimonic well-being,” which represents the personal and social abilities that contribute to a person’s optimal psychological functioning, such as believing in the meaning of life, a sense of continued personal growth, and social contribution (Ryff, 2018). These two kinds of well-being have different theoretical bases and philosophical traditions; therefore, it is necessary to distinguish between them when discussing the relationship between self-control, motivation, and happiness (Delle Fave, 2014; Joshanloo et al., 2020). Second, the relationship between self-control and happiness is closely related to one’s real-life conditions; however, this study focused on college students who live on campuses and have less complicated life experiences than most adults. Therefore, future research should focus on adult populations from various backgrounds. Third, both motivation and happiness have different cultural meanings. Thus, there are a large number of cross-cultural studies that indicate that it is necessary to explore the relationship between self-control, motivation, and happiness from a cultural perspective. Fourth, our research was confined to the laboratory setting. Considering the ecological validity of this strand of research, future studies should be carried out in ecological contexts (Steger, 2016; Joshanloo et al., 2020), to explore the more realistic relationships among these variables and provide effective suggestions for improving peoples’ happiness.

## Conclusion

The present study found that self-control has a positive effect on subjective well-being and that this effect is mainly moderated by prevention motivation. Specifically, when prevention motivation increases, an individual is more likely to have a positive subjective well-being due to the resulting higher levels of self-control. Regarding the state aspect, ego depletion reduces one’s level of subjective well-being; however, ego depletion does not have a completely linear relationship with subjective well-being as a moderator of motivation, especially among people with prevention motivation (both trait and state). When the two types of prevention motivation are consistent, ego depletion has the most obvious impact on subjective well-being. Our research suggests that there are limitations on the association between self-control and happiness and that motivation should be the focus of future studies. Through long-term motivation training or providing temporary situational stimulation to a person’s motivation, the effect of self-control on one’s subjective well-being can be influenced, implying that these methods have practical value for improving people’s happiness.

## Data Availability Statement

The raw data supporting the conclusions of this article will be made available by the authors, without undue reservation.

## Ethics Statement

The studies involving human participants were reviewed and approved by the Ethics in Human Research Committee of the School of Psychology, Northwest Normal University. The patients/participants provided their written informed consent to participate in this study.

## Author Contributions

GZ designed the experiments. QZ and WW recruited the participants and collected the data. FX and YC performed the data analyses. GZ, FX, YCL, and YXL wrote the manuscript. All authors contributed to the article and approved the submitted version.

## Conflict of Interest

The authors declare that the research was conducted in the absence of any commercial or financial relationships that could be construed as a potential conflict of interest.

## Publisher’s Note

All claims expressed in this article are solely those of the authors and do not necessarily represent those of their affiliated organizations, or those of the publisher, the editors and the reviewers. Any product that may be evaluated in this article, or claim that may be made by its manufacturer, is not guaranteed or endorsed by the publisher.
